# Effects of Light Quality and Phytochrome Form on Melatonin Biosynthesis in Rice

**DOI:** 10.3390/biom10040523

**Published:** 2020-03-30

**Authors:** Ok Jin Hwang, Kiyoon Kang, Kyoungwhan Back

**Affiliations:** 1 Department of Biotechnology, College of Agricultural and Life Sciences, Chonnam National University, Gwangju 61186, Korea; smilax@jnu.ac.kr; 2 Division of Life Sciences, College of Life Sciences and Bioengineering, Incheon National University, Incheon 22012, Korea; kykang@inu.ac.kr

**Keywords:** cadmium, blue light, light intensity, melatonin, rice *phy* mutants

## Abstract

Light is an important factor influencing melatonin synthesis in response to cadmium treatment in rice. However, the effects of light quality on, and the involvement of phytochrome light receptors in, melatonin production have not been explored. In this study, we used light-emitting diodes (LEDs) to investigate the effect of light wavelength on melatonin synthesis, and the role of phytochromes in light-dependent melatonin induction in rice. Upon cadmium treatment, peak melatonin production was observed under combined red and blue (R + B) light, followed by red (R) and blue light (B). However, both far-red (FR) LED light and dark treatment (D) failed to induce melatonin production. Similarly, rice seedlings grown under the R + B treatment showed the highest melatonin synthesis, followed by those grown under B and R. These findings were consistent with the results of our cadmium treatment experiment. To further confirm the effects of light quality on melatonin synthesis, we employed rice photoreceptor mutants lacking functional phytochrome genes. Melatonin induction was most inhibited in the phytochrome A mutant (*phyA*) followed by the *phyB* mutant under R + B treatment, whereas *phyB* produced the least amount of melatonin under R treatment. These results indicate that PhyB is an R light receptor. Expression analyses of genes involved in melatonin biosynthesis clearly demonstrated that tryptophan decarboxylase (*TDC*) played a key role in phytochrome-mediated melatonin induction when rice seedlings were challenged with cadmium.

## 1. Introduction

Melatonin acts as a potent free radical scavenger and antioxidant in almost all organisms, including animals and plants [1,2,3,4]. Melatonin acts as a hormone in plants, and as a signaling molecule in a wide range of biological activities [5]. For example, melatonin treatment enhances plant tolerance against many stresses, including salt [6,7], drought [8,9], viruses [10], pathogens [11,12], waterlogging [13], and senescence [14], among others [15,16,17]. Melatonin is also involved in plant development processes such as growth [18,19], seed viability [20], flowering [21,22], endoplasmic reticulum (ER) quality control [23,24], secondary metabolite synthesis [25], and others [26]. The pleiotropic effects of melatonin are due to the combined effects of its antioxidant activity and signaling or hormonal activity [5], although its metabolites, such as 2-hydroxymelatonin and cyclic 3-hydroxymelatonin, are also involved in physiological activity such as tiller growth [27,28].

In plants, melatonin biosynthesis involves four enzymatic steps, beginning with the catalyzation of tryptophan decarboxylase (TDC), the first committed-step enzyme, into tryptamine. The second enzyme is tryptamine 5-hydroxylase, a P450 enzyme that converts tryptamine into serotonin. The penultimate enzyme is serotonin *N*-acetyltransferase (SNAT), which is responsible for *N*-acetylserotonin (NAS) synthesis. The final enzyme is *N*-acetylserotonin *O*-methyltransferase (ASMT), which catalyzes NAS into melatonin [29]. In each step, multiple isogenes participate in precursor synthesis; for this reason, it is difficult to generate melatonin knockout plants. Due to the low catalytic efficiency of the final two enzymes (SNAT and ASMT), plant melatonin levels are extremely low; therefore, precise quantification of melatonin levels remains challenging, even with the use of cutting-edge liquid chromatography–mass spectrometry (LC–MS) analytical tools [14,30]. However, plant melatonin synthesis can be induced in response to various stimuli, including pathogen infection and cadmium treatment [30,31]. Among many candidates, cadmium is optimal for eliciting melatonin synthesis in rice [32]; however, it cannot induce melatonin production under dark conditions [33]. Therefore, unlike melatonin synthesis in animals, plant melatonin synthesis is dependent on light [34]. In this study, we investigated the effect of light wavelength dependence on melatonin production in rice with and without cadmium treatment, as well as the involvement of phytochromes in this process.

## 2. Materials and Methods

### 2.1. Cadmium Treatment

Rice (*Oryza sativa*) seeds were sterilized and grown on half-strength Murashige and Skoog (MS) medium under a 12 h light (60 μmol × m^−2^ × s^−1^)/12 h dark photoperiod at 28 °C/24 °C (day/night) for 7 days. We incubated 20 seedlings for 3 days in a 50-mL conical tube, in water containing 0.5 mM CdCl_2_ (Sigma-Aldrich, St. Louis, MO, USA) at 28 °C under different light sources: combined monochromic red and blue (R + B) light (7:3 ratio, 60 μmol × m^−2^ × s^−1^), red (R, 60 μmol × m^−2^ × s^−1^), blue (B, 60 μmol × m^−2^ × s^−1^), or far-red light (FR, 4 μmol × m^−2^ × s^−1^). Leaves and stems were harvested for further analyses. The light was provided via light-emitting diodes (LEDs; STECH LED, Gyeonggi-do, Korea).

### 2.2. Semi-Quantitative Reverse Transcription–Polymerase Chain Reaction (RT-PCR) Analysis

Total RNA was isolated from rice plants using a NucleoSpin RNA Plant Kit (Macherey-Nagel, Düren, Germany). First-strand cDNA was synthesized from 2 μg total RNA using MG MMLV reverse transcriptase (MGmed, Seoul, Korea) and an oligo dT_18_ primer at 42 °C for 1 h. Semi-quantitative RT-PCR was performed as described previously [33].

### 2.3. Quantification of Serotonin, N-Acetylserotonin, and Melatonin

For serotonin and NAS measurements, 0.1 g frozen samples were pulverized into powder in liquid nitrogen using the TissueLyser II system (Qiagen, Tokyo, Japan) and extracted with 1 mL methanol for 1 h at room temperature. Samples were then centrifuged for 10 min at 12,000 × *g*, and each supernatant (20 µL) was subjected to high-performance liquid chromatography (HPLC) using a fluorescence detection system (Waters, Milford, MA, USA). For melatonin measurements, 0.1 g samples were pulverized into powder in liquid nitrogen and extracted with 1 mL chloroform for 16 h at 4 °C. Chloroform extracts (200 µL) were completely evaporated and dissolved in 0.1 mL 40% methanol; 20 µL aliquots were injected into the HPLC system, which included a fluorescence detector (Waters). All measurements were performed in triplicate.

### 2.4. Statistical Analysis

Means were compared using analysis of variance (ANOVA) with IBM SPSS Statistics software (IBM, Armonk, NY, USA, version 25.0), followed by post-hoc Tukey’s honest significant difference (HSD) tests. A *P*-value < 0.05 was taken to indicate statistical significance. All data are presented as means ± standard deviation.

## 3. Results

### 3.1. R + B Treatment Induced Peak Melatonin Synthesis upon Cadmium Treatment

**Figure 1:**
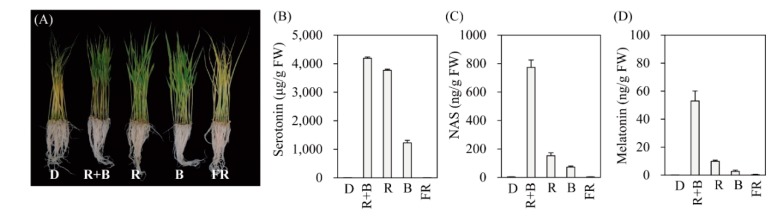
Effects of various light wavelengths on serotonin, *N*-acetylserotonin, and melatonin production in rice seedlings. (**A**) Phenotypes, (**B**) serotonin, (**C**) *N*-acetylserotonin, and (**D**) melatonin levels in response to cadmium treatment under various light conditions. Seven-day-old rice seedlings were rhizosperically challenged with 0.5 mM CdCl_2_ for 3 days under continuous light wavelength condition. D, dark; R + B, combined red and blue LED light (7:3 ratio); R, red LED light; B, blue LED light; FR, far-red LED light. Light intensity in R + B, R, and B was equal to 60 μmol × m^−2^ × s^−1^ whereas FR was 4 μmol × m^−2^ × s^−1^. Values are means ± standard deviations of three independent experiments. FW, fresh weight.

To determine the light wavelength most effective for inducing melatonin production, 7-day-old rice seedlings were challenged with cadmium for 3 days under four different LED wavelengths. As shown in Figure 1, melatonin production was not induced in response to cadmium under the dark treatment (D). Light-triggered melatonin production was highest under the R + B treatment, followed by the R and B treatments (Figure 1D). FR produced melatonin levels comparable to those of the D treatment. Similar to melatonin, its precursors serotonin and NAS were also maximally induced by R + B, followed by R and B, whereas no induction was observed under the D or FR treatments (Figure 1B,C). These data strongly suggest that melatonin induction upon cadmium treatment is completely dependent on specific light wavelengths. Maximum melatonin production under R + B treatment appears to be closely associated with the spectrum of photosynthetically active radiation, since R + B produces a higher photosynthetic rate than the sum of R and B alone [35].

### 3.2. Melatonin Levels in Rice Seedlings Grown under Various Light Conditions

**Figure 2:**
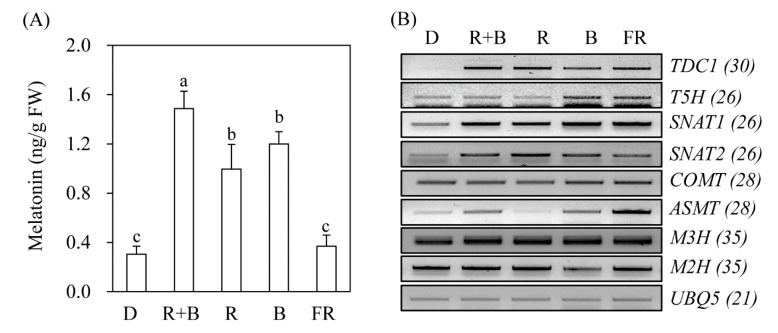
Effects of light wavelength on melatonin synthesis and expression levels of corresponding genes. (**A**) Effects of light quality on melatonin synthesis. (**B**) Expression levels of melatonin biosynthetic and catabolic genes. Dehusked rice seeds were germinated and grown in half-strength Murashige and Skoog (MS) medium for 7 days under continuous light conditions as described in Figure 1. *TDC1*, tryptophan decarboxylase 1; *T5H*, tryptamine 5-hydroxylase; *SNAT*, serotonin *N*-acetyltransferase; *COMT*, caffeic acid *O*-methyltransferase; *ASMT*, *N*-acetylserotonin *O*-methyltransferase; *M3H*, melatonin 3-hydroxylase; *M2H*, melatonin 2-hydroxylase, *UBQ5*, ubiquitin 5. Values are means ± standard deviations of three independent experiments. FW, fresh weight. Different letters denote significant differences as determined by Tukey’s post hoc HSD test at *P* < 0.05. Numbers in parentheses are the numbers of polymerase chain reaction (PCR) cycles. GenBank accession numbers are AK069031 (*TDC1*), AK071599 (*T5H*), AK059369 (*SNAT1*), AK068156 (*SNAT2*), AK064768 (*COMT*), AK072740 (*ASMT1*), AK067086 (*M3H*), AK119413 (*M2H*), and Os03g13170 (*UBQ5*).

To determine whether melatonin production is dependent on light wavelength even in the absence of elicitors such as cadmium, rice seeds were germinated in MS medium and grown under various light conditions. As shown in Figure 2, rice seedlings grown under the D treatment produced about 0.3 ng/g fresh weight (FW) melatonin, which was identical to that of the FR treatment. In contrast, rice seedlings grown under R + B light produced about 1.5 ng/g FW melatonin, which was 5-fold higher than that of the D treatment, confirming that melatonin production is light-dependent. The melatonin production of rice seedlings grown under R and B alone was about 1 ng/g FW. The key gene responsible for light-dependent melatonin induction appears to be *TDC* (Figure 2B). *TDC* mRNA expression was induced under all light conditions, including FR, but not under the D treatment. Similarly, *SNAT1* and *SNAT2* expression increased under all light conditions compared to that of the D treatment. *ASMT* expression varied significantly among light conditions. The expression level of melatonin 3-hydroxylase (*M3H*), which catalyzes melatonin into cyclic 3-hydroxymelatonin, remained unchanged, whereas that of melatonin 2-hydroxylase (*M2H*), which catalyzes melatonin into 2-hydroxymelatonin, was increased under R + B, R, and FR light conditions, and decreased under B light conditions. Based on these results, it was difficult to identify the specific genes responsible for melatonin production under various light wavelengths.

### 3.3. Phytochrome-Dependent Melatonin Production

**Figure 3:**
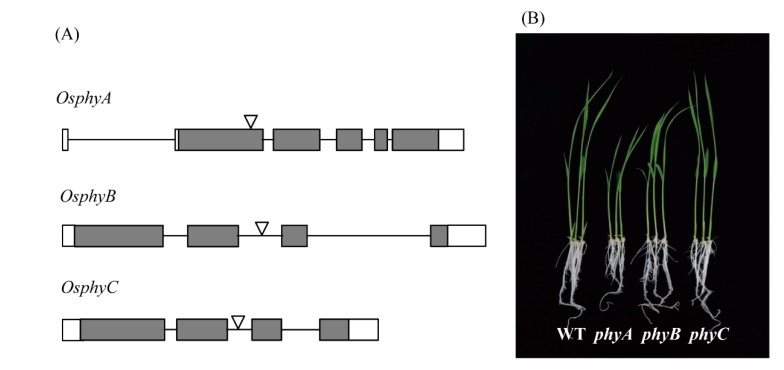
T-DNA insertional lines of rice *Phy* genes and their seedling phenotypes. (**A**) Simple schematic diagrams of rice *Phy* gene structures and T-DNA insertion sites [37]. (**B**) Phenotypes of 7-day-old rice seedlings grown in half-strength MS medium under a 12 h light/12 h dark cycle at 28 °C. Exons (filled boxes), introns (lines), and 5′- and 3′-untranslated regions (open boxes) are shown. Reverse triangles indicate T-DNA insertion sites.

**Figure 4:**
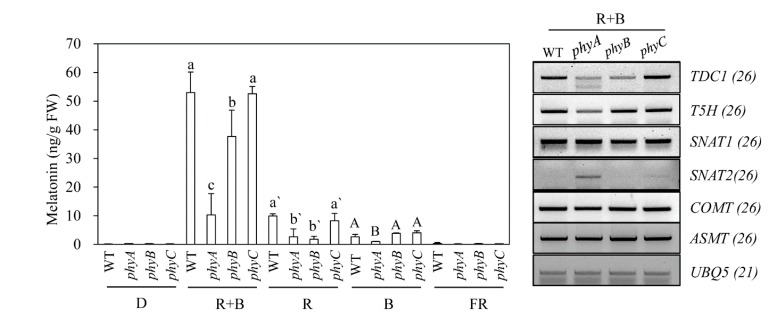
Effects of various light wavelengths on melatonin synthesis in *phy* mutants. (**A**) Melatonin levels in response to cadmium treatment under various light conditions in *phy* mutants. (**B**) Expression levels of melatonin biosynthetic genes. Seven-day-old rice seedlings were rhizosperically challenged with 0.5 mM CdCl_2_ for 3 days under various light wavelength condition as described in Figure 1. Values are means ± standard deviations of three independent experiments. FW, fresh weight. Different letters denote significant differences as determined by Tukey’s post hoc HSD test at *P* < 0.05. GenBank numbers are presented in Figure 2.

Rice contains three types of phytochromes (PhyA, PhyB, and PhyC) that perceive R and FR light [36]. To determine whether light-dependent melatonin production is associated with light receptor phytochrome, we employed rice phytochrome (*phy*) mutants to see whether these mutants show impaired melatonin production under various light conditions [37,38]; these mutants were obtained from the Crop Biotech Institute at Kyung Hee University (Seoul, Korea) [39]. T-DNA insertion sites and phenotypes of 7-day-old seedlings of three *phy* mutants are shown in Figure 3. Following cadmium challenge under various light conditions, melatonin production was most severely inhibited in the *phyA* mutant under R + B treatment, followed by *phyB*, whereas melatonin production in the *phyC* mutant remained similar to that of the wild-type (WT) (Figure 4A). Under R treatment, melatonin production was most reduced in the *phyB* mutant, followed by the *phyA* mutant, suggesting that PhyB is the major R light receptor. Similar to the R + B condition, the *phyC* mutant produced comparable amounts of melatonin to WT under the R condition. In response to the B treatment, melatonin synthesis was significantly diminished only in the *phyA* mutant, whereas *phyB* and *phyC* showed no difference in melatonin synthesis compared to WT. Thus, PhyA predominantly receives B light. The role of PhyC appears to be negligible in terms of light perception and melatonin production in response to cadmium treatment. Notably, melatonin levels were much higher under R + B treatment compared to the sum of the R and B treatments alone.

To identify a key regulatory gene among the *phy* mutants responsible for melatonin production, we monitored the expression of melatonin biosynthetic and catabolic genes under R + B treatment. The severe reduction in melatonin production observed in the *phyA* mutant was closely associated with lower expression levels of *TDC* and caffeic acid *O*-methyltransferase (*COMT*) (Figure 4B), which are key genes for light-dependent melatonin synthesis in response to cadmium treatment [33].

### 3.4. Effects of Light Intensity on Serotonin and Melatonin Production under R + B Treatment

**Figure 5:**
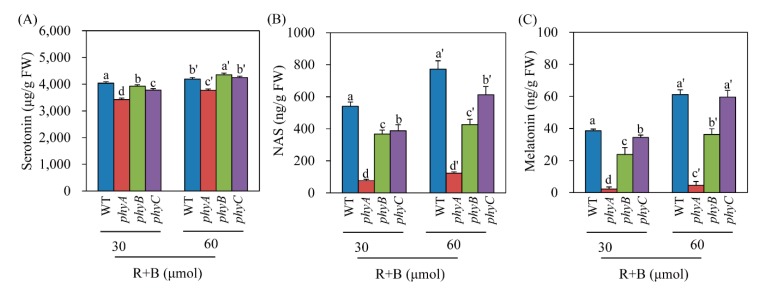
Effects of light intensity on (**A**) serotonin, (**B**) *N*-acetylserotonin, and (**C**) melatonin levels in *phy* mutants in response to cadmium treatment under two light intensity conditions. Seven-day-old rice seedlings were rhizosperically challenged with 0.5 mM CdCl_2_ for 3 days under two different continuous light intensity conditions. Light intensity (R + B) was either 30 or 60 μmol × m^−2^ × s^−1^. Values are means ± standard deviations of three independent experiments. FW, fresh weight. Different letters denote significant differences as determined by Tukey’s post hoc HSD test at *P* < 0.05.

Serotonin is the only essential precursor for melatonin biosynthesis in plants [29]. In plants, the synthesis of both serotonin and melatonin is dependent on light (Figure 1) [40]. To determine whether light intensity affected the levels of serotonin, NAS, and melatonin in the *phy* mutants under R + B treatment, we measured these compounds in R + B treatments with two different light intensity values: 30 and 60 μmol × m^−2^ × s^−1^. Serotonin levels were indistinguishable between the light intensity treatments among WT and the *phyB* and *phyC* mutants; however, the *phyA* mutant had lower serotonin levels (Figure 5A). In contrast, NAS production increased as light intensity increased in WT and the *phyC* mutant, whereas no difference was observed in the *phyA* or *phyB* mutant, suggesting that these mutants failed to perceive light intensity differences (Figure 5B). As observed for melatonin, the WT and *phyC* mutant responded to increased light intensity by enhancing melatonin production (Figure 5C). However, *phyA* completely lost its ability to increase melatonin production as light intensity increased. Although *phyB* responded to light intensity, its responsiveness was lower than that of WT and the *phyC* mutant. These results indicate that *phyA* cannot perceive light as well as light intensity, resulting in a severe decrease in melatonin production in response to cadmium treatment under the R + B condition.

### 3.5. Seed Melatonin Levels in Healthy phy Mutants

**Figure 6:**
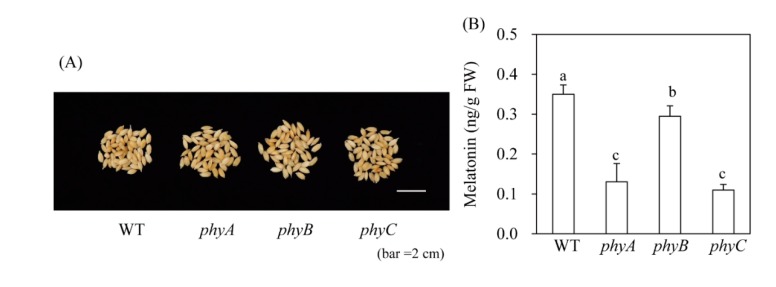
Seed melatonin levels in *phy* mutants. (**A**) Water-imbibed rice seeds after 9 h. (**B**) Melatonin levels in *phy* mutants. WT, wild type; FW, fresh weight. Different letters indicate significant differences, as determined by Tukey’s honest significant difference (HSD) post-hoc test at a level of *P* < 0.05.

Melatonin production in response to cadmium treatment may not accurately reflect in vivo plant melatonin levels. Thus, to confirm our findings, we quantified melatonin levels in rice seeds, which have a slightly higher melatonin content than other tissues such as green leaves; seeds also contain far fewer compounds that interfere with the HPLC chromatogram than green leaves. WT rice seeds contained 0.4 ng/g FW melatonin, which was significantly more than that of the *phy* mutant (Figure 6). For example, *phyA* and *phyC* contained 0.12 ng/g FW melatonin and *phyB* contained 0.3 ng/g FW melatonin. These in vivo data were slightly different from our cadmium treatment data. In response to cadmium treatment, the *phyC* mutant and WT induced melatonin synthesis to a similar extent; in seeds, melatonin levels decreased to a similar extent in the *phyA* and *phyC* mutants. Conversely, *phyB* produced less melatonin than WT under cadmium treatment, and in seeds. Although different melatonin responses were observed between control seeds and cadmium-treated seedlings among the three *phy* mutants, our results clearly demonstrate that melatonin synthesis is dependent on light, as well as phytochrome light receptors.

## 4. Discussion

### 4.1. Biological Significance of the Dependency of Plant Melatonin Synthesis on Light

Unlike melatonin in animals, plant melatonin levels are higher under high-light versus low-light conditions [41]. Melatonin levels in *Glycyrrhiza uralensis* roots were first discovered to be higher under R light than under B or white light, indicating that light wavelength may influence plant melatonin levels [42]. The production of melatonin and its metabolite N^1^-acetyl-N^2^-formyl-5-methoxykynuramine (AFMK) has shown a rhythmic pattern, peaking during the late light phase in water hyacinth [43]. These findings indicate that light intensity and light wavelength are pivotal factors in plant melatonin production. This hypothesis was confirmed by Lee et al. [33], who reported that melatonin induction required light when rice seedlings were challenged with cadmium; their detailed mechanistic study demonstrated that cadmium treatment promoted an oxidative burst, characterized by increased H_2_O_2_ and NO production, which triggered melatonin synthesis. Due to the close relationship between the oxidative burst and melatonin induction, it is highly likely that plant melatonin is involved in regulating the cellular reactive oxygen species (ROS) balance in both normal healthy cells and stressed cells [16]. Our observation that melatonin synthesis increased under R + B treatment compared to the sum of R and B alone strongly implies an intimate relationship between melatonin and photosynthesis, because photosynthesis shows far greater enhancement under R + B treatment relative to the sum of the R and B treatments alone [34]. Under light conditions, chloroplasts are a major catalyst of ROS production due to partial reduction of O_2_ in plants. Light-harvesting complexes, such as photosystem I and II (PSI and PSII, respectively) and the electron transport chain (ETC), can generate a range of ROS including superoxide (O_2_^–^), hydrogen peroxide (H_2_O_2_), and singlet oxygen (^1^O_2_), which is generated from chloroplasts and triggers membrane lipid peroxidation, leading to growth inhibition and even cell death by damaging the PSII reaction center [44]. In response to abiotic stresses, such as drought and high light conditions, chloroplasts produce more ROS, such that more ROS scavengers are required, including antioxidants and antioxidant enzymes [44]. The close relationship between high photosynthesis activity and peak melatonin synthesis under the R + B condition has a genetic basis; the *Arabidopsis* melatonin-deficient *snat1* knockout mutant showed enhanced susceptibility to high light stress [45]. In addition, chloroplasts are major sites for melatonin biosynthesis, and contain the penultimate SNAT enzyme and key regulatory enzymes, such as M2H and *N*-acetylserotonin deacetylase [46,47,48]. Thus, melatonin plays an important role in maintaining chloroplast integrity by upregulating melatonin synthesis during photosynthesis, or in response to abiotic stresses, to efficiently scavenge ROS and induce ROS-scavenging enzymes [5].

### 4.2. Photoreceptor-Mediated Melatonin Production in Plants

Plants perceive light signals through a number of photoreceptors including phytochromes, cryptochromes, phototropins, and UVR8. Phytochromes absorb the R/FR region of the light spectrum (600–750 nm), whereas cryptochromes and phototropins absorb B/ultraviolet light (280–500 nm). UVR8 is involved in sensing ultraviolet light in the 280–315 nm range, and is highly involved in plant stress responses [49]. Based on melatonin synthesis induction via light and ROS in plants [33], it is clear that all three photoreceptors are necessary for plant melatonin synthesis. Among the three photoreceptors, phytochromes have been extensively studied due to their involvement in a vast array of plant biological functions, including germination, de-etiolation, the shade-avoidance response, photosynthesis, photomorphogenesis, and senescence [50]. Phytochromes exist in two interconvertible forms, namely inactive Pr and active Pfr; R light induces inactive Pr to become active Pfr, whereas FR light transforms active Pfr into inactive Pf. Active Pfr is translocated into the nucleus, where it mediates various transcriptional activities. Rice contains three types of phytochromes (PhyA, PhyB, and PhyC); PhyA and PhyB perceive R light in a redundant manner, whereas PhyA is involved in perceiving FR light [51]. In the context of phytochrome functions and melatonin synthesis, we predicted that the R treatment would perform best in terms of inducing melatonin synthesis, if phytochromes were exclusively involved in melatonin induction in response to cadmium treatment. However, R + B treatment, rather than R, yielded the highest melatonin production, at 50 ng/g FW, which was 5-fold higher than that of the R treatment (Figure 1D). Consistent with this result, melatonin induction was most significantly inhibited under R + B treatment in the *phyA* mutant, followed by *phyB* rather than *phyC*. In response to B treatment, the *phyA* mutant failed to induce melatonin synthesis, whereas melatonin synthesis was not reduced in the *phyB* and *phyC* mutants. This result suggests that, in the context of melatonin synthesis in rice, PhyA can perceive B light. Thus, phytochromes are required for melatonin synthesis, by absorbing light for photosynthesis and thus inducing the generation of a series of ROS, which are key signaling molecules for melatonin synthesis induction in response to cadmium treatment. The active Pfr and inactive Pr interconvertible phytochrome forms are not directly responsible for melatonin synthesis; rather, phytochromes are indirectly involved in this process through light absorption. The most important wavelengths for photosynthesis are R (600–700 nm) and B light (420–450 nm), which coincide well with chlorophyll absorption peaks. Collectively, the wavelengths for maximum melatonin synthesis are in exact agreement with those for maximum photosynthesis, suggesting that melatonin acts as a master regulator, enhancing photosynthesis by scavenging ROS generated within chloroplasts [5]. This conclusion is consistent with the findings of many previous studies, which reported that melatonin deficiency leads to growth inhibition [52,53] and premature senescence [14].

## 5. Conclusions

Unlike animals, plants require light for melatonin synthesis. However, no studies have clarified the roles of light quality and photoreceptors in plant melatonin biosynthesis. In this study, we investigated the optimum wavelengths for melatonin synthesis in rice, as well as the role of phytochromes in this process. We treated rice seedlings with cadmium to induce melatonin synthesis under light at various wave lengths and found that R + B treatment enhanced melatonin production to a greater extent than the sum of the R and B treatments alone. This is the first study to report that phytochromes are involved in melatonin production by absorbing R + B light, rather than in a Pf/Pfr light-dependent manner. The maximum melatonin production under R + B treatment was similar to the optimum photosynthesis rate under R + B treatment, suggesting that melatonin is functionally coupled with photosynthesis, possibly due to its potent antioxidant activity and role in inducing antioxidant enzymes.
